# The *Phe362Tyr* mutation conveying resistance to organophosphates occurs in high frequencies in salmon lice collected from wild salmon and trout

**DOI:** 10.1038/s41598-017-14681-6

**Published:** 2017-10-27

**Authors:** Helene Børretzen Fjørtoft, Francois Besnier, Anne Stene, Frank Nilsen, Pål Arne Bjørn, Ann-Kristin Tveten, Bengt Finstad, Vidar Aspehaug, Kevin Alan Glover

**Affiliations:** 1 Norwegian University of Science and Technology, Department of Biological Sciences in Aalesund, P.O. Box 1517, N-6025 Aalesund, Norway; 2 University of Bergen, Department of Biology, Sea Lice Research Center, P.O. Box 7803, N-5020 Bergen, Norway; 3 Institute of Marine Research, P.O. Box 1870, N-5817 Bergen, Norway; 4 Norwegian Institute for Nature Research, P.O. Box 5685, N-7485 Trondheim, Norway; 5 PatoGen Analyse AS, P.O. Box 1527, N-6025 Aalesund, Norway

## Abstract

The parasitic salmon louse, and its resistance to chemical delousing agents, represents one of the largest challenges to the salmon aquaculture industry. We genotyped lice sampled from wild salmon and sea trout throughout Norway with the recently identified *Phe362Tyr* mutation that conveys resistance to organophosphates. These results were compared to data from lice sampled on farmed salmon in the same regions. The resistant (R) allele was observed in salmon lice from wild salmon and sea trout throughout Norway, although its frequency was highest in farming-intense regions. In most regions, the frequency of the R allele was higher in lice collected from wild sea trout than wild Atlantic salmon, and in all regions, the frequency of the R allele was similar in lice collected from wild sea trout and farmed Atlantic salmon. The R allele is only selected for in fish-farms where organophosphates are used for delousing. Therefore, our results suggest extensive exchange of lice between farmed and wild hosts, and indicate that in farming-dense regions in Norway, aquaculture represents a major driver of salmon louse population structure. Finally, these data suggest that the wild hosts within the regions studied will not delay the spread of resistance when organophosphates are used.

## Introduction

Commercial aquaculture of salmonids in sea-cages started in Norway in the early 1970’s^1^. From a modest start with 100 tons of Atlantic salmon (*Salmo salar*) and 540 tons of rainbow trout (*Oncorhynchus mykiss*) produced in 1971, the Norwegian aquaculture industry harvested 1.25 million tons in 2014^1,2^. The industry has grown to be economically highly significant in several countries, and global production of Atlantic salmon exceeded 2.3 million tons in 2014^3^. However, the rapid expansion of marine cage-based salmon aquaculture has not been without environmental impacts. These include escapees that may genetically interact with wild conspecifics^4^, benthic sediment changes under farms^5^, and potential transmission of pathogens, such as the parasitic salmon louse (*Lepeophtheirus salmonis*), between farmed and wild salmonids^6–9^.

Salmon aquaculture is primarily based upon rearing fish to market size in open sea-cages located in coastal areas. These traditional cages provide farmed fish with no protection from pathogens found in the surrounding water masses, including the naturally occurring salmon louse. The salmon louse is an ectoparasitic copepod that feeds on mucus, skin and blood of its salmonid hosts^10,11^. It displays eight life stages: three free-living planktonic stages until it settles on a host, followed by two non-mobile chalimus stages, while the last stages, two preadult and one adult, are mobile^12^. During the free-living stages lice are passively transported by the water currents, but move vertically in response to light and salinity^13^. The two nauplii stages last approximately forty degree-days at water temperatures of 10 °C, after which they moult into the infectious copepodid stage that is able to attach to a host^14^. The copepodid stage lasts approximately 130 degree-days at the same temperature, until the energy reserve from the egg is spent^14,15^. A model taking into account water currents and temperature has estimated that salmon lice may drift up to 200 kilometres from hatching until the energy is spent, but that most travel less than 25 kilometres before they reach the point at which they need to have attached to a host^16^.

Already by the mid-1970s, epizootics of salmon lice were reported on salmon held in farms in both Norway and Scotland^17,18^. Although a wide range of delousing methods have been implemented since these early reports^8^, the salmon louse remains as a major challenge to cage-based aquaculture of salmonids. The total cost of dealing with this parasite for the Norwegian aquaculture industry alone was estimated at 351 million dollars in 2014^19^. The Norwegian Atlantic salmon production constituted 56% of the world’s total production in 2014^3^. Assuming the same salmon louse cost per ton salmon produced worldwide, a rough estimate of the total cost of salmon lice is 645 million dollars.

The increase in salmon aquaculture has led to a major increase in the number of hosts available to salmon lice in the coastal zone throughout the year. Consequently, there is a considerable production of salmon lice larvae from farms^20–24^ which comes in addition to the natural production on wild salmonid hosts, even though lice levels are generally held low on farms due to stringent legislation^8,20^. The consequence is that in aquaculture intense regions, lice reproducing on farmed salmon can represent a major component of the total salmon louse population^20^. Consistent with this is the fact that epizootics of salmon lice have been observed on wild sea trout (*Salmo trutta*), Atlantic salmon and Arctic char (*Salvelinus alpinus*) in and near farming-dense regions^25–28^. Furthermore, several studies have reported an association between distance to nearest farm and the number of lice on wild salmonids^24,29–31^.

The organophosphates trichlorphon and dichlorvos were the only delousing chemicals used in commercial aquaculture in Norway until the early 1990s, when hydrogen peroxide was used for a short period, but they were not fully dethroned before pyrethroids were introduced in the mid-1990s^17,32–35^. Frequent use of a chemotherapeutant drives selection for individuals that can survive the treatment^35^. In 1992, the first cases of reduced sensitivity towards dichlorvos were documented^36^. In the same year, a new organophosphate, azamethiphos, was introduced^37^. However, it soon became clear that salmon lice displaying resistance to one organophosphate also were resistant to others^38^. By the end of the 1990s, other delousing compounds, such as pyrethroids and emamectin benzoate were preferred, but as decreased sensitivity towards the new chemicals also developed, organophosphates were re-introduced^35,39^. In 2008, azamethiphos was used again in Norway after almost a decade, but already the next year treatment failures were reported^35,39^. Nevertheless, in 2014, 749 prescriptions, or 21% of the total delousing prescriptions in Norway, were given for azamethiphos^40^. Organophosphates target acetylcholinesterase (AChE) by blocking the cleavage of acetyl choline^41^. Recently, a point mutation in the ace genes of the salmon louse, the *Phe362Tyr* mutation, was identified and linked to organophosphate resistance in this parasite^39^. The ace genes encode AChE, and mutations here are associated with organophosphate resistance in several arthropods^42^. The *Phe362Tyr* mutation can occur as heterozygote (RS) or as homozygote (RR), inducing intermediate or fully resistant genotypes, respectively^39^.

The salmon louse, and resistance to chemical delousing agents, represents the primary challenge to the continued development of an economically viable and environmentally sustainable salmonid aquaculture production^9^. Currently, resistance has been reported on all available delousing compounds in Norway, except from flubenzurones^40^. The use of the latter is however strictly regulated due to potential negative effects on the environment^43^. Unlike farmed salmonids, wild salmonids are never directly exposed to delousing compounds. Therefore, it is theoretically possible that lice attached to wild salmonids could serve as a refuge for chemical-sensitive lice, and contribute to delay the development of resistance in aquaculture^44,45^. Consequently, there is a need to elucidate genetic relationships and connectivity among lice sampled from wild and farmed hosts in time and space, identify the mechanisms by which chemical resistance is dispersed within and among regions, and finally, to investigate whether wild salmonids may serve as a refuge for chemical-sensitive lice. To address these issues, we genotyped lice collected from wild salmon and sea trout in Norway in 2014 using the recently identified mutation *Phe362Tyr* ^39^. The frequencies of the genotypes (RR, RS and the fully sensitive SS) on lice collected from wild fish were thereafter compared to the frequencies observed on farmed salmonids in the same regions and time-scale.

## Results

### General patterns

**Figure 1:**
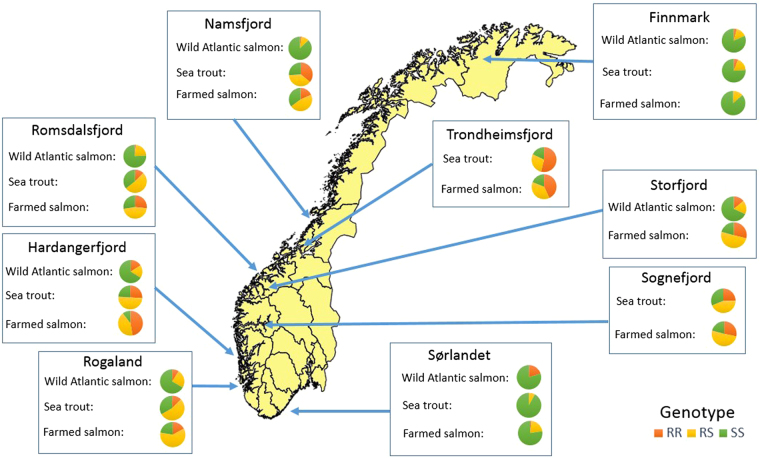
The prevalence of the genotypes SS, RS and RR in salmon lice (*L. salmonis*) sampled from wild Atlantic salmon, sea trout and farmed Atlantic salmon in Norway in 2014. For Finnmark, only 2013 farm data were available. The background map is derived from Global Administrative Areas^67^ and R packages^68–71^.

At all locations sampled along the coast, both wild Atlantic salmon and sea trout were infected with salmon lice carrying the mutation *Phe362Tyr*, which is associated with reduced sensitivity towards organophosphates (i.e., RS or RR genotypes) (Fig. 1, Supplementary Table 1). This coincides with the fact that the R allele was present in lice from all farm sites sampled in Norway in the same period (Supplementary Table 1)^46^.

The RR genotype was present in all samples of lice from wild salmonids, with the exception of the sample taken from wild sea trout at Sørlandet, southern Norway. It was also missing in farmed Atlantic salmon in Finnmark, northern Norway. The heterozygote genotype was present in all samples of lice from wild and farmed hosts. The lowest prevalence of the RS genotype was found in lice from the sea trout sample from Sørlandet (8%), while the highest prevalence was found in lice from farmed salmon in Rogaland (60%). The fully sensitive homozygote genotype SS was also present in all samples of lice. Both Finnmark and Sørlandet have a prevalence of more than 75% SS in all datasets sampled in these regions, which are outside the intense farming regions.

### Background data from farms

The dataset, based upon the raw data presented in Kaur and colleagues^46^ on farmed salmon from 2014, demonstrates a very low prevalence of RR in Finnmark (0%, 2013 data) and Sørlandet(1.7%). The highest prevalence of RR (47%) was found in the Hardangerfjord. The heterozygote genotype RS exceeded 50% prevalence in Storfjord, Sognefjord and Rogaland, while the lowest prevalence in farmed salmon was found in Finnmark (13.6%).

In Kaur and colleagues^46^, data from 2013 are also included (Supplementary Figure 1, Supplementary Table 2). Four of the regions included in this study have data from both years, and all have significant changes in genotype frequencies between 2013 and 2014. For Namsfjord (*P* < 0.001) the change is towards less resistance, while for Trondheimsfjord (*P* < 0.001), Sognefjord (*P* = 0.01) and Hardangerfjord (*P* < 0.001) the prevalence of the resistant genotypes increases.

### Wild sea trout

In the lice collected from sea trout, the RR genotype was rare in Finnmark (6%) and missing in Sørlandet. In mid-Norway, it was frequent (Fig. 1). The highest prevalence of the RR genotype was from lice collected on sea trout captured in Trondheimsfjord (54%). The RS genotype was rare in Sørlandet (8%), while it was common in most other regions. In Romsdalsfjord, Hardangerfjord and Rogaland, 50% or more of the salmon lice from sea trout displayed the RS genotype. Salmon lice sampled from sea trout had high prevalence of the genotype SS in Sørlandet (92%) and Finnmark (76%), while the other regions had prevalences ranging from 35.6% in Romsdalsfjord to 18% in Trondheimsfjord. The genotype frequencies in salmon lice sampled from sea trout from different regions differed significantly from each other (*P* < 0.001).

### Wild Atlantic salmon

In the lice collected from wild Atlantic salmon, the RR genotype was found in all regions, but with a prevalence below 5% in Finnmark, Namsfjord and Romsdalsfjord. The prevalence of RR was highest (16%) in the samples collected from wild Atlantic salmon in Hardangerfjord. The prevalence of the heterozygote genotype RS ranged from 10.6% in Namsfjord to 24.4% in Rogaland. SS prevalence ranged from 87.2% in Namsfjord to 66% in Hardangerfjord. The genotype frequencies of salmon lice sampled from wild Atlantic salmon in different regions varied significantly (*P* < 0.01). Both within each region and for the total wild Atlantic salmon sample, we found no difference in genotype frequencies between salmon lice sampled from 1 sea winter (sw), 2 sw or 3 sw hosts (Supplementary Figure 1). *P*-values are given in the Supplementary file.

### Comparisons between species and wild vs farmed

**Figure 2:**
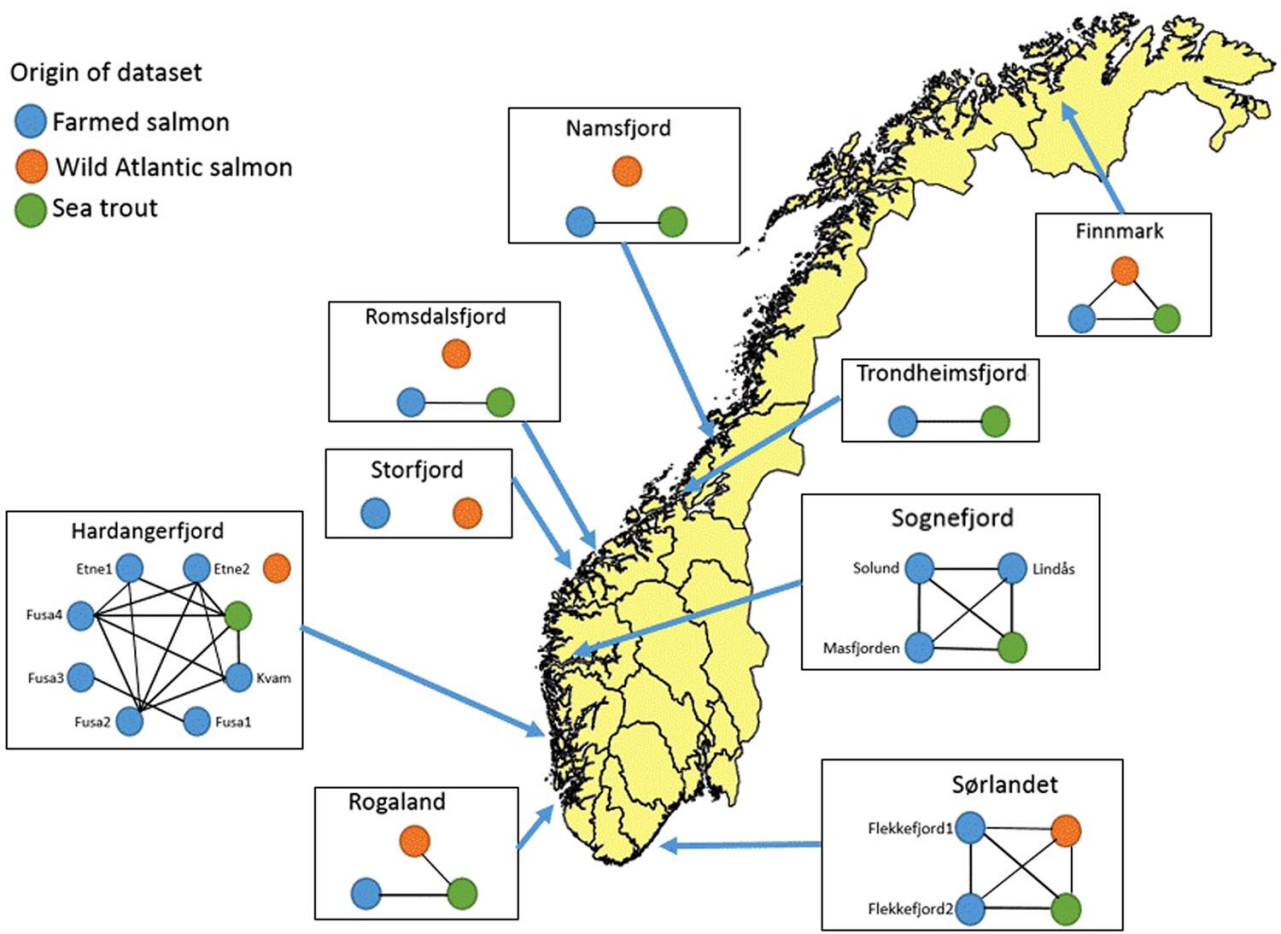
Illustration of similarities in genotype frequencies between salmon lice collected on wild salmon, wild sea trout, and farmed salmon in 2014, by region. For Finnmark, only 2013 farm data were available. Where there is no significant difference between two subsets (*P* > 0.00081, Bonferroni corrected), the two are connected with a line. If two subsets are significantly different, there is no line between them. The background map is derived from Global Administrative Areas^67^ and R packages^68–71^.

The results of the comparisons of genotype frequencies between lice collected on the different host groups are summarized (Fig. 2, *P*-values given in the Supplementary file). In Finnmark, Rogaland and Sørlandet, no significant difference in genotype frequencies were observed between lice collected from wild Atlantic salmon and sea trout (Supplementary Table 4). In the middle regions Namsfjord, Romsdalsfjord and Hardangerfjord, genotype frequencies differed significantly between lice collected on wild salmon and on sea trout (*P* < 0.00081 in all three regions). Here, lice collected on sea trout displayed higher frequencies of the R allele than lice collected on wild salmon.

In Sognefjord and Sørlandet, the different farm datasets did not differ significantly from each other or from the local sea trout datasets. In Hardangerfjord, two out of seven farm datasets were significantly different from the other farm and wild datasets, but not from each other (Supplementary Table 5). In these two datasets, the frequencies of the R allele are very high. The sea trout dataset from Hardangerfjord is not significantly different from the remaining farm datasets (Supplementary Tables 4 and 5). In the remaining regions with only one farm dataset, there are no significant differences between the local sea trout datasets and the farm datasets (Supplementary Table 4).

In Finnmark and Sørlandet, the far north and the far south, the datasets from farmed and wild salmon did not differ significantly from each other (Supplementary Table 4). In Namsfjord, Romsdalsfjord, Storfjord, Hardangerfjord and Rogaland, lice collected on wild Atlantic salmon displayed significantly lower frequencies of the genotypes including the *Phe362Tyr* mutation compared to all the farm data sets from their respective regions (all *P*-values < 0.00081).

### Hardy-Weinberg equilibrium (HWE)

Six of the datasets from wild and farmed salmonids deviated significantly from HWE. The wild Atlantic salmon datasets from Storfjord, Hardangerfjord and Sørlandet all have deficit RS frequencies (*P* < 0.001, *P* < 0.001 and *P* = 0.002, respectively). The farmed salmon subset Etne2 has deficit RS (*P* < 0.001), while the subset Etne1 on the contrary has an excess RS frequency (*P* = 0.002). The farmed salmon subset from Rogaland also has an excess heterozygote allele frequency (*P* = 0.008).

## Discussion

This is the first study to investigate the frequency of the *Phe362Tyr* mutation, which conveys resistance towards organophosphates, in salmon lice collected on wild salmonids. The most significant results can be summarized as follows: 1. In all regions studied in Norway, lice displaying the organophosphate resistance allele (R) were observed on both wild salmon and sea trout, 2. In most of these regions, the frequency of the R allele was higher in lice collected from wild sea trout than in lice collected on wild Atlantic salmon, 3. In all of these regions, the genotype frequency was similar in samples of lice collected from wild sea trout and farmed Atlantic salmon, 4. In most of these regions, the frequency of the R allele was lower in samples of lice collected from wild compared to farmed salmon, 5. The frequency of the R allele was highest in lice collected from wild salmon and sea trout in farming dense regions.

The R allele in salmon lice, conveying resistance to organophosphates, is inadvertently selected for in fish-farms where organophosphates are used to delouse infected fish. Although the origin of the individual parasites genotyped in this study could not be determined (nor was this our intention), our data demonstrate exchange of salmon lice between farmed and wild hosts. Furthermore, given that the lice sampled from sea trout in this study displayed a very similar frequency of the R allele to lice sampled on farmed salmon in all regions investigated, we suggest that within farming-dense regions in Norway, aquaculture represents a significant driver of the salmon louse infection dynamics over time. This is consistent with the results of other studies that have demonstrated that infection levels observed on wild salmonids are closely linked with distance to nearby farms^24,29–31,47^. Finally, our data suggest that there is no natural refuge for sea lice displaying complete sensitivity to organophosphates within the regions studied, as the R allele is present also in lice from wild hosts and in areas without the use of organophosphates. The latter conclusion is consistent with population genetic studies that have demonstrated that there is a high degree of gene-flow among lice from both sides of the Atlantic^48–51^.

Resistance is expected to develop when a population is repeatedly subject to a chemical treatment over time^35^. Organophosphates were the first treatments used on commercial fish-farms against salmon lice, and were used extensively for almost two decades before alternatives were introduced. During this period, lice underwent strong selection towards resistance^35^. When organophosphates were reintroduced after almost a decade’s absence in 2008, new reports of resistance followed immediately^39^, indicating that the allele conveying resistance to organophosphates was present in salmon lice also in the period in which this chemical was not used. In a recent study by Kaur and colleagues^52^, the frequency of salmon lice carrying the R allele in Norwegian fish-farms during the period when organophosphates were not used are reported (see raw data in the paper’s supplementary information). In 1998, when organophosphate usage was minimized, 50% of the sampled lice carried the mutation^52^. In 2000 the prevalence was 20%, 31% in 2002, and 8% and 13% in southern and northern Norway, respectively, in 2009^52^. Interestingly, the RR genotype is only present, in low frequency (5%), in the sample from 1998, and is completely missing in the samples from 2000, 2002 and 2009^52^. The reduction in the prevalence of the R allele in the period without selection pressure may be due to dilution from wild hosts and a fitness cost associated with being homozygote resistant. However, the continued presence of heterozygote lice indicate that any cost associated with this genotype is too small to completely remove the R-allele from the population even after a decade without exposure to chemical treatment. Hardy-Weinberg equilibrium (HWE) tests can identify whether a sample includes an excess or deficit of a certain genotypic class, i.e., homo or heterozygotes, in relation to the frequency of alleles in the sample. This, among other things, can potentially identify selection. While we observed several deviations from HWE in the samples of lice collected from wild salmon, no systematic or consistent deviations were observed. Thus, the degree to which a potential fitness-cost has influenced genotype frequencies in the wild samples collected here remains unresolved. Despite this, one farm sample displayed a strong deficit of RS heterozygotes and a high frequency of RR individuals. This farm had been treated with organophosphates three times during the period 2012–2014, and the RS deficiency is potentially explained by a high selection pressure on individuals carrying the RR genotype.

Salmon lice residing on wild salmonids are not directly exposed to chemicals, and are therefore not subject to selection towards resistance. Our findings of high frequencies of both homozygote and heterozygote resistant salmon lice collected on wild salmonids supports the theory that over a period of time, aquaculture has represented a major source of the salmon lice settling on wild salmonids in regions with intense aquaculture. In these areas, we believe that repeated treatments have selected for individuals carrying the resistant allele, and this trait has become settled in salmon lice in those regions due to connectivity between farms and between lice on farmed and wild hosts^16,23,53^. Salmon lice descending from such regions are thus likely to carry the R-allele irrespective of their host. In the geographic scope of this study, this translates to all regions with the exception of Sørlandet and the far northern region Finnmark. In all of these regions, the genotype frequencies of salmon lice from farmed salmon and from sea trout are not significantly different, implying that they have the same source.

In most regions studied, lice collected from wild sea trout displayed a higher frequency of the R allele than lice collected from the wild salmon. We propose that this contrast between lice collected from the two wild host species reflects key differences in their ecology. Specifically, the use of the coastal feeding grounds for sea trout vs. the oceanic feeding grounds for salmon. Sea trout are most abundant in the fjords and coastal zone during the summer months, with a peak in activity from May through July^54^. Both post-smolts and older individuals have a strong coastal preference, and rarely move more than 80 km from their native river^55–57^. These are the same coastal areas where salmon farms are typically located. Therefore, given the sea trout´s ecology, they will be exposed to infection pressure from the same source of salmon lice as farmed salmon. This was demonstrated in the present study as the genotype frequency on lice collected from sea trout matched that of lice collected on farmed salmon in the same region.

In contrast to sea trout, Atlantic salmon smolts migrate through fjords and coastal areas to the feeding areas in the North Atlantic where they typically stay 1–3 years before they return to their native river to spawn^58^. Wild Atlantic salmon that have stayed more than one year at sea display salmon lice at all developmental stages when they return to the coast, as do salmon caught at sea^59–61^. This means that salmon returning to rivers after spending 1–3 years in the sea may have been infected in coastal areas as post-smolts on their seaward migration (same as for sea trout), on the oceanic feeding grounds (unlike sea trout), and finally, in the coastal region once again upon their return migration (same as sea trout). One potential explanation to the observed lower frequencies of the resistance allele on lice from wild salmon as opposed to sea trout, could have been that the salmon lice on wild Atlantic salmon represent the situation in the fjord when the wild salmon left as post-smolts, and that the frequency of the R allele in lice in aquaculture-dense regions increased in the period that the wild salmon were at sea. However, we found no significant difference in genotype frequencies between samples from wild salmon hosts that had spent one, two or three years at sea, neither within each region, nor in the total wild salmon sample. On the oceanic feeding grounds, salmon from different regions and countries intermix^62,63^. Therefore, we suggest that the observed lower frequency of the R allele on wild salmon in this study is to a certain degree caused by wild salmon being infected with salmon lice on the oceanic feeding grounds. These lice may have originated from outside farming dense regions where the frequency of the R allele is likely to be lower due to a lack of farming induced evolution (e.g. far north and south of Norway, Denmark, parts of UK, Russia). Indeed, cross-infection between salmon from geographically divergent areas, while on the high seas, has been proposed as a major mechanism by which the salmon louse displays a high level of genetic exchange across geographical distances where lice larvae cannot drift^48–51^. It is nevertheless important to point out that as salmon are not continuously exposed to salmon lice directly from fish farms while on the oceanic feeding grounds, in contrast to coastal-living sea trout, the potential role of selection (i.e., lower fitness) against RR or RS genotypes cannot be excluded as a potential contributory factor for the observed lower frequency of the R allele on lice sampled from wild salmon.

It has been speculated whether wild salmonids may act as a refuge for chemotherapeutant sensitive salmon lice^44,45^. Based upon the strong concordance in the R allele frequency on wild sea trout and farmed salmon within the same region, we conclude that sea trout cannot be regarded as a refuge for salmon lice sensitive to organophosphates in regions with intensive aquaculture. The situation is slightly different for wild Atlantic salmon within the same aquaculture-dense regions, as they may also be infected with lice on the oceanic feeding grounds that originate from outside aquaculture-dense regions, with an associated lower frequency of the R allele. However, to what degree low density or non-farming regions can be regarded as a significant refuge for sensitive lice is questionable. Population genetic studies of salmon lice across the north Atlantic have demonstrated a high degree of gene-flow^48–51^. Importantly, an analysis of >5000 single nucleotide polymorphism markers on lice collected from throughout the North Atlantic demonstrated that reduced sensitivity to an alternative chemotherapeutant used for delousing, emamectin benzoate, originated at one location and was rapidly spread to all sampled regions in the north Atlantic within just 10–40 generations (~3–5 generations/year)^51^. The frequency of the R allele varies regionally and with time (Kaur and colleagues^46,52^, our results), presumably driven by the intensity of chemical usage locally. After a period with frequent use of organophosphates from 2008 to 2014, we document high frequencies of the R allele in salmon lice from both wild and farmed hosts, and the RR genotype is again present in almost all samples. Reduced use of organophosphates in the future due to the increased use of non-chemical delousing methods will probably reduce the frequency of the R allele in the salmon lice population again, as was observed in the period when organophosphates were replaced by other compounds in Norway^40,52^. Our findings of resistant alleles (R), and in particular the fully resistant genotype (RR), in salmon lice from an area (Sørlandet) and hosts that have not been exposed to organophosphate treatments support the previous findings of high connectivity between salmon lice in geographically distinct areas. These results suggest that lice residing on wild salmonids, either within or outside farming-dense regions, will not serve as a major source of sensitive lice once resistance is already well-established and not as long as the use of organophosphates is continued. Nevertheless, lice collected from wild salmonids far outside farming regions need to be studied to validate this.

## Materials and Methods

### Sampling lice on wild salmon and sea trout

**Figure 3:**
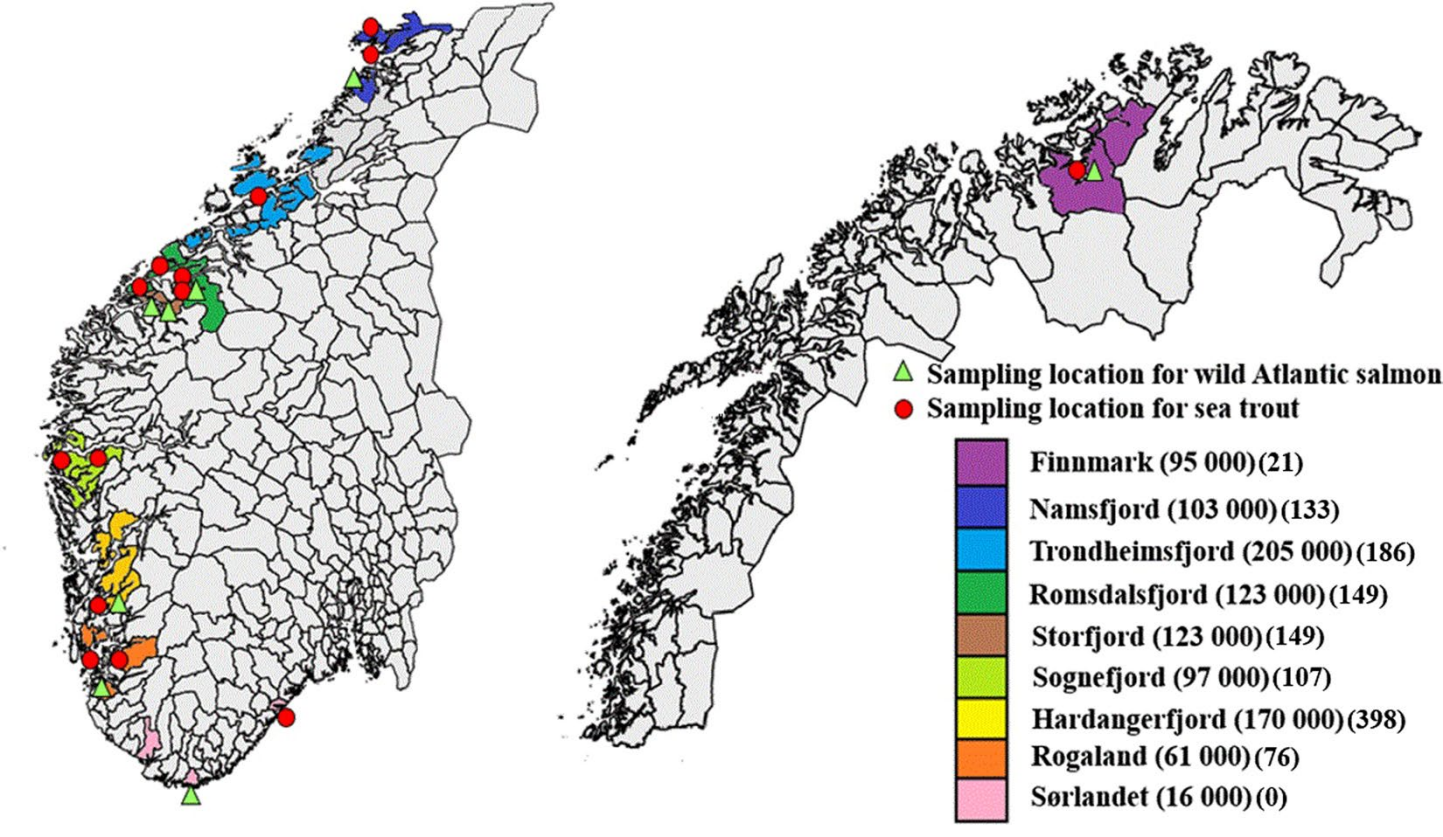
Overview of the regions included in the study. Numbers in the first parenthesis represent how many tons of farmed Atlantic salmon that were slaughtered in the relevant counties in 2014^2^, while the second parenthesis sums the number of organophosphate treatments at county level for the years 2012–2014^65^. The background map is derived from Global Administrative Areas^67^ and R packages^68–71^.

Since 1992, the Norwegian national salmon lice monitoring program has registered infection levels on wild salmonids captured in different parts of the country on an annual basis^64^. Our study used the sampling areas established by this program; Altafjord in Finnmark, Namsfjord, Trondheimsfjord and Romsdalsfjord in Mid-Norway, Sognefjord, Hardangerfjord and Rogaland in Western Norway, and a control area, Sørlandet, in Southern Norway (Fig. 3). Sørlandet has marginal aquaculture production and no chemicals were used in this region in 2012–2014^65^. In Finnmark, in the far north, salmon lice numbers in aquaculture have been low, but started increasing in 2013^65^. Organophosphates were not used in Finnmark in 2012, but in 2013 three sites were treated, and ten sites in 2014^65^. The number of organophosphate treatments for the period 2012–2014 are reported at county level in Fig. 3, and at municipality level for the relevant sampling locations in Table 1 in the Supplementary file. Sea trout were captured at multiple locations in all eight regions, and wild salmon in six of them (Supplementary Table 1). An additional wild salmon sampling region was established in Storfjord, Mid-Norway (Fig. 3, Supplementary Table 1).

In each region, salmon lice from sea trout were sampled by the National monitoring program for salmon lice. The program is initiated by The Norwegian Food Safety Authority and The Ministry of Trade, Industry and Fisheries, and the permission to capture wild sea trout is given by the Norwegian Environment Agency. In the river Etne located in the Hardangerfjord, sea trout and wild salmon were caught in an upstream fish trap, and salmon lice were sampled by trained personnel. The fish trap is located approximately 900 meters from the river mouth. There are no rapids or major obstacles between the sea and the trap that would slow the ascending fish. The fish trap project was led by the Institute of Marine Research, and the permission to capture wild salmonids was given by the Norwegian Environment Agency. Salmon lice from coastal migrating wild Atlantic salmon at all other locations were obtained in cooperation with local bag net fishermen. The bag net fishery is strictly regulated, and all participants have a National authorization. The fish were killed with a sharp blow to the head immediately after capture. When the fish was landed, salmon lice were sampled either by the fishermen or by trained personnel. Our project did not have a license to capture wild salmonids, but was able to reuse or utilize samples of wild salmonids that were captured by other ongoing activities along the coast, all of which have specific permits for sampling and catching salmonids as described above. The salmon louse is not subject to The European Convention for the protection of Vertebrate Animals used for Experimental and other Scientific Purposes or by Norwegian legislation.

From each wild host, up to ten salmon lice were collected and stored in one container filled with 70% ethanol. The container was marked with the ID of the host, date and location. A scale sample was also taken from the host, along with length, weight and the total number of salmon lice. Scale samples were used to test whether the captured Atlantic salmon were wild or farmed escapees^66^, and how long they had been at sea. Scale samples are unfortunately missing from the region Finnmark. Salmon lice from hosts that were of farm origin were not included in the study. All samples were taken in the period May-August 2014.

In total, 301 salmon lice sampled from wild Atlantic salmon at nine locations and 523 salmon lice from sea trout at sixteen locations are included in this study. A full overview of the dataset is given in Supplementary Table 1.

### Genotyping lice collected on wild salmon and sea trout

All lice sampled on wild salmon and sea trout, as described above, were genotyped by PatoGen Analyse AS (Aalesund/Norway), using their patented TaqMan assay for identification of the mutation *Phe362Tyr*. The assay classifies each louse as fully sensitive homozygotes (SS), partly sensitive heterozygotes (RS), or resistant homozygotes (RR). This is the exact same assay used by Kaur and colleagues^46^ to investigate frequencies of resistant and sensitive lice on farmed salmonids in Norway.

### Background data from farms

The SS, RS and RR allele-frequency distributions for lice collected on wild salmonids were compared to a comprehensive data set of genotype frequencies for lice collected on farmed salmon in the same regions and period. Data from the farmed fish were not generated in the present study, but were taken from a recent publication, which reported allele frequencies at the municipality level^46^. We used the raw data, which is available in the publication’s S1 Table under Supporting Information^46^.

In order to select the background data that were relevant to our study, we used the border of the municipality where we had sampling stations and set a perimeter of 50 kilometers based on the dispersal distances of salmon lice and sea trout^16,53,57^. If the perimeter crossed the border of a municipality reported in Kaur and colleagues^46^, we included their data in our study (Fig. 3). Because we do not know which farm within the given region that was sampled, the true distance from our sampling site to the farm location may be greater than 50 kilometers in some cases. In Southern Norway, the 50 kilometer perimeter criteria was not applicable. The only farms included in Kaur and colleagues^46^ are located in the westernmost part of the region, while the National monitoring program’s wild salmonid sampling location is in the easternmost part of this region, ~190 kilometers away.

All lice from wild salmonids were sampled in 2014, while Kaur and colleagues^46^ analyzed lice sampled on farmed salmon in the period 2012–2014. For the comparison between salmon lice from wild and farmed hosts, we used data from the farmed fish in 2014, except for the region Finnmark where background data from farms only were available from 2013 (Fig. 1). In the large study of Kaur and colleagues^46^, no significant difference in genotype frequencies were found between years. However, as we selected data from some regions, we re-tested this material for differences in the frequencies of the genotypes between lice collected in 2013 and 2014 in instances where both were available. Based upon these selection criteria, a subset of 3438 salmon lice from 17 farm locations are included for 2014, and 704 salmon lice from twelve farm locations for 2013 (Supplementary Tables 1 and 2).

### Statistics

In each region, a pair-wise comparison between the genotype frequencies of lice collected from the different host types (farmed salmon, wild salmon, wild sea trout) were conducted using chi-square tests. *H0* was no difference in genotype frequencies between subsets from different hosts within a region. In regions where multiple farm sites were included, these were not pooled, but instead tested for differences. A total of 62 tests were performed. By applying a Bonferroni correction, the 5% threshold value was set to *P = *0.00081.

The genotype frequencies of salmon lice from sea trout from different regions were compared using a chi-square test (*df* = 14), and the same test was performed for salmon lice from wild Atlantic salmon (*df* = 12). The genotype frequencies of salmon lice from 1 sw, 2 sw and 3 sw wild Atlantic salmon were compared using chi square statistics, both within each region and for the total wild Atlantic salmon sample (*df* = 4 for all tests).

In the regions Namsfjord, Trondheimsfjord, Sognefjord and Hardangerfjord we have background data from salmon lice on farms for both 2013 and 2014. The pooled 2013 data were compared to the pooled 2014 data within each of the regions using chi square tests (*df* = 2).

A Hardy-Weinberg test was conducted to check for significant departure from Hardy-Weinberg equilibrium within each set of samples. The farm data was not pooled within a region when more than one dataset was available.

## Electronic supplementary material

Supplementary PDF File
