# Supplementary material for: The Phe362Tyr mutation conveying resistance to organophosphates occurs in high frequencies in salmon lice collected from wild salmon and trout
Source: Sci Rep. 2017 Oct 27;7:14258. doi: 10.1038/s41598-017-14681-6 (PMC5660183; doi:10.1038/s41598-017-14681-6)
Supplement: Supplementary file 1 — Supplementary PDF File [file 41598_2017_14681_MOESM1_ESM.pdf]

Supplementary file

The *Phe362Tyr* mutation conveying resistance to organophosphates occurs in high frequencies in salmon lice collected from wild salmon and trout.

Helene Børretzen Fjørtoft[1,2*], Francois Besnier[3], Anne Stene[1], Frank Nilsen[2], Pål Arne Bjørn[3], Ann-Kristin Tveten[1], Bengt Finstad[4], Vidar Aspehaug[5], Kevin Alan Glover[2,3].

Supplementary Table 1: Distribution of genotypes in salmon lice (*L. salmonis*) sampled from wild and farmed salmonids in Norway in 2014 and 2013 (Finnmark). All data labelled "Farmed salmon" are acquired from a paper by Kaur and colleagues (2016)[46], while "wild salmon" and "sea trout" are our own data. In regions with data from more than one municipality, the results for the whole region are also presented. The number of treatments for farmed salmon are for the specific farms sampled, given by Kaur and colleagues (2016)[46]. For wild salmon and sea trout, the number is for total organophosphate treatments within the municipality of the sampling location in the same period[65].

Region	Municipality	No of locations	Host	Year	N	Genotype			Treatments 2012-2014
						RR	RS	SS	
Finnmark	Kvalsund	1	Farmed salmon	2013	118	0,0 %	13,6 %	86,4 %	0
Finnmark	Alta	1	Wild salmon	2014	49	4,1 %	14,3 %	81,6 %	4
Finnmark	Alta	2	Sea trout	2014	50	6,0 %	18,0 %	76,0 %	4
Namsfjord	Flatanger	1	Farmed salmon	2014	180	18,3 %	46,7 %	35,0 %	4
Namsfjord	Flatanger	1	Wild salmon	2014	47	2,1 %	10,6 %	87,2 %	20
Namsfjord	Vikna	2	Sea trout	2014	50	36,0 %	38,0 %	26,0 %	46
Trondheimsfjord	Hemne	1	Farmed salmon	2014	174	43,1 %	37,9 %	19,0 %	1
Trondheimsfjord	Hitra/Agdenes	2	Sea trout	2014	50	54,0 %	28,0 %	18,0 %	54/1
Romsdalsfjord	Midsund	1	Farmed salmon	2014	142	26,8 %	45,8 %	27,5 %	2
Romsdalsfjord	Rauma	2	Wild salmon	2014	50	2,0 %	22,0 %	76,0 %	1
Romsdalsfjord	Haram/Fræna/Molde/Rauma	4	Sea trout	2014	177	12,4 %	52,0 %	35,6 %	4/1/2/1
Storfjord	Skodje	1	Farmed salmon	2014	329	28,9 %	50,5 %	20,7 %	1
Storfjord	Ørskog/Stordal	2	Wild salmon	2014	50	14,0 %	18,0 %	68,0 %	0/0
Sognefjord	Solund	1	Farmed salmon	2014	168	30,4 %	51,8 %	17,9 %	2
Sognefjord	Lindås	1	Farmed salmon	2014	118	18,6 %	59,3 %	22,0 %	4
Sognefjord	Masfjorden	1	Farmed salmon	2014	183	32,8 %	44,3 %	23,0 %	2
Sognefjord	Whole region	3	Farmed	2014	469	28,4 %	50,8 %	20,9 %	8
Sognefjord	Gulen/Høyanger	2	Sea trout	2014	48	25,0 %	43,8 %	31,3 %	6/10
Hardangerfjord	Kvam	1	Farmed salmon	2014	243	37,0 %	50,6 %	12,3 %	1
Hardangerfjord	Fusa	4	Farmed salmon	2014	1094	55,6 %	38,4 %	6,0 %	11
Hardangerfjord	Etne	2	Farmed salmon	2014	357	28,6 %	48,7 %	22,7 %	3
Hardangerfjord	Whole region	7	Farmed salmon	2014	1694	47,2 %	42,3 %	10,5 %	15
Hardangerfjord	Etne	1	Wild salmon	2014	50	16,0 %	18,0 %	66,0 %	12
Hardangerfjord	Etne	1	Sea trout	2014	50	26,0 %	50,0 %	24,0 %	12
Rogaland	Tysvær	1	Farmed salmon	2014	152	17,1 %	60,5 %	22,4 %	1
Rogaland	Sandnes	1	Wild salmon	2014	45	8,9 %	24,4 %	66,7 %	2
Rogaland	Tysvær/Hjelmeland	2	Sea trout	2014	48	12,5 %	54,2 %	33,3 %	11/5
Sørlandet	Flekkefjord	2	Farmed salmon	2014	298	1,7 %	20,8 %	77,5 %	0
Sørlandet	Mandal	1	Wild salmon	2014	10	20,0 %	0,0 %	80,0 %	0
Sørlandet	Risør	1	Sea trout	2014	50	0,0 %	8,0 %	92,0 %	0

Supplementary Table 2: Distribution of genotypes in salmon lice (*L. salmonis*) sampled from farmed salmon in Norway in 2013. Data from Kaur and colleagues (2016)[46].

Region	Municipality	No of locations	Host	Year	N	Genotype			Treatments 2012-2014
						RR	RS	SS	
Finnmark	Kvalsund	1	Farmed salmon	2013	118	0,0 %	13,6 %	86,4 %	0
Namsfjord	Vikna	1	Farmed salmon	2013	61	83,6 %	16,4 %	0,0 %	0
Namsfjord	Nærøy	1	Farmed salmon	2013	60	26,7 %	73,3 %	0,0 %	2
Namsfjord	Flatanger	1	Farmed salmon	2013	36	16,7 %	66,7 %	16,7 %	0
Trondheimsfjord	Bjugn	1	Farmed salmon	2013	78	28,2 %	56,4 %	15,4 %	4
Trondheimsfjord	Snillfjord	1	Farmed salmon	2013	90	23,3 %	33,3 %	43,3 %	1
Trondheimsfjord	Kristiansund	1	Farmed salmon	2013	12	16,7 %	41,7 %	41,7 %	0
Trondheimsfjord	Averøy	1	Farmed salmon	2013	54	7,4 %	14,8 %	77,8 %	0
Trondheimsfjord	Halsa	1	Farmed salmon	2013	30	33,3 %	53,3 %	13,3 %	1
Sognefjord	Hyllestad	1	Farmed salmon	2013	40	47,5 %	45,0 %	7,5 %	0
Hardangerfjord	Kvinnherad	1	Farmed salmon	2013	85	43,5 %	28,2 %	28,2 %	0
Hardangerfjord	Kvinnherad	1	Farmed salmon	2013	60	10,0 %	56,7 %	33,3 %	0

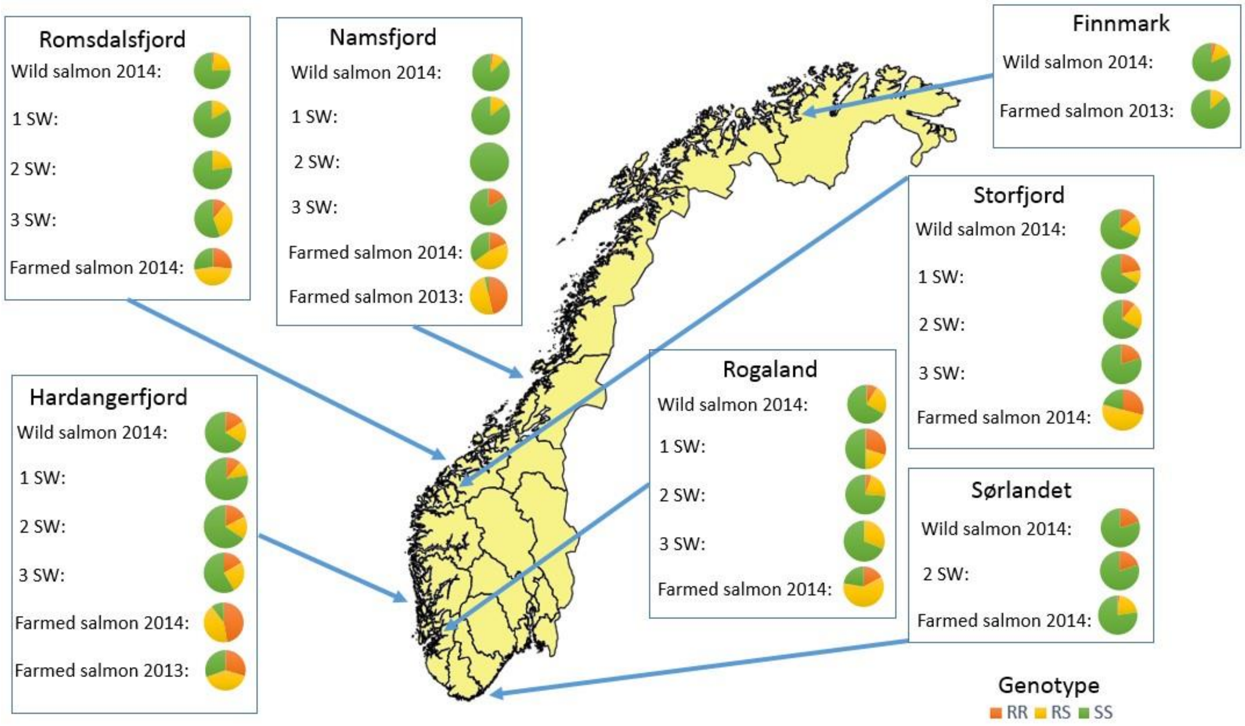

Supplementary Figure 1: Genotype frequencies of salmon lice sampled from different cohorts of wild Atlantic salmon in 2014 and from farmed salmon in 2013 and 2014. There are no significant differences between 1 sw, 2 sw or 3 sw wild Atlantic salmon samples within any region. The genotype frequencies in salmon lice from farmed salmon are significantly different between years. The background map is derived from Global Administrative Areas[67] and R packages[68,69,70,71].

Supplementary Table 3: Statistics for chi-square test of salmon lice from 1 sw, 2 sw and 3 sw wild Atlantic salmon.

Region	Chi-square	df	p-value
Namsfjord	8.68	4	0.069
Romsdalsfjord	5.1	4	0.27
Storfjord	2.3	4	0.66
Hardangerfjord	1.0	4	0.90
Rogaland	7.75	4	0.10
All regions	1.6076	4	0.807

Supplementary Table 4: Comparison of genotype frequencies between wild Atlantic salmon, sea trout and farmed salmon samples of salmon lice, by region. *P*-values in red are significant according to the Bonferroni corrected threshold value, 0.00081.

Region	Wild salmon vs sea trout	Wild salmon vs farmed salmon	Sea trout vs farmed salmon
Finnmark	0.78222	0.08528	0.01827
Namsfjord	1.8309e-09	2.41975e-10	0.0283
Trondheimsfjord			0.3491
Romsdalsfjord	2.03185e-06	5.16464e-09	0.00441
Storfjord		1.69624e-11	
Sognefjord			
Farmed Solund			0.13109
Farmed Lindås			0.18678
Farmed Masfjorden			0.40587
Hardangerfjord	9.51607e-05		
Farmed Kvam		4.22888e-16	0.0682
Farmed Fusa 1		6.20603e-39	1.48016e-10
Farmed Fusa 2		1.5809e-08	0.44305
Farmed Fusa 3		1.21887e-45	4.54132e-10
Farmed Fusa 4		2.88311e-10	0.32498
Farmed Etne 1		3.60946e-09	0.24413
Farmed Etne 2		1.35642e-07	0.11465
Rogaland	0.00485	1.7151e-07	0.28823
Sørlandet	0.00423		
Farmed Flekkefjord 1		0.00087	0.08323
Farmed Flekkefjord 2		0.00235	0.05519

Supplementary Table 5: Comparison of genotype frequencies between samples of salmon lice from different farms in the Hardangerfjord in 2014. *P*-values in red are significant according to the Bonferroni corrected threshold value, 0.00081.

Farm	Kvam	Fusa 1	Fusa 2	Fusa 3	Fusa 4	Etne 1	Etne 2
Kvam	1	1.25688e-10	0.60961	1.0630353e-09	0.686238	1.0571961e-05	0.0012861
Fusa 1	*	1	1.058726e-08	0.546413	1.093823e-09	2.078828e-26	1.325982e-13
Fusa 2	*	*	1	5.88480e-08	0.957263	0.006127	0.065667
Fusa 3	*	*	*	1	7.6175733e-09	1.22690e-26	4.7677474e-15
Fusa 4	*	*	*	*	1	0.0031761	0.017333
Etne 1	*	*	*	*	*	1	2.239640e-07
Etne 2	*	*	*	*	*	*	
